# Supplementary material for: A versatile Plasmodium falciparum reporter line expressing NanoLuc enables highly sensitive multi-stage drug assays
Source: Commun Biol. 2023 Jul 12;6:713. doi: 10.1038/s42003-023-05078-5 (PMC10338434; doi:10.1038/s42003-023-05078-5)
Supplement: Supplementary file 2 — Supplementary Information [file 42003_2023_5078_MOESM2_ESM.pdf]

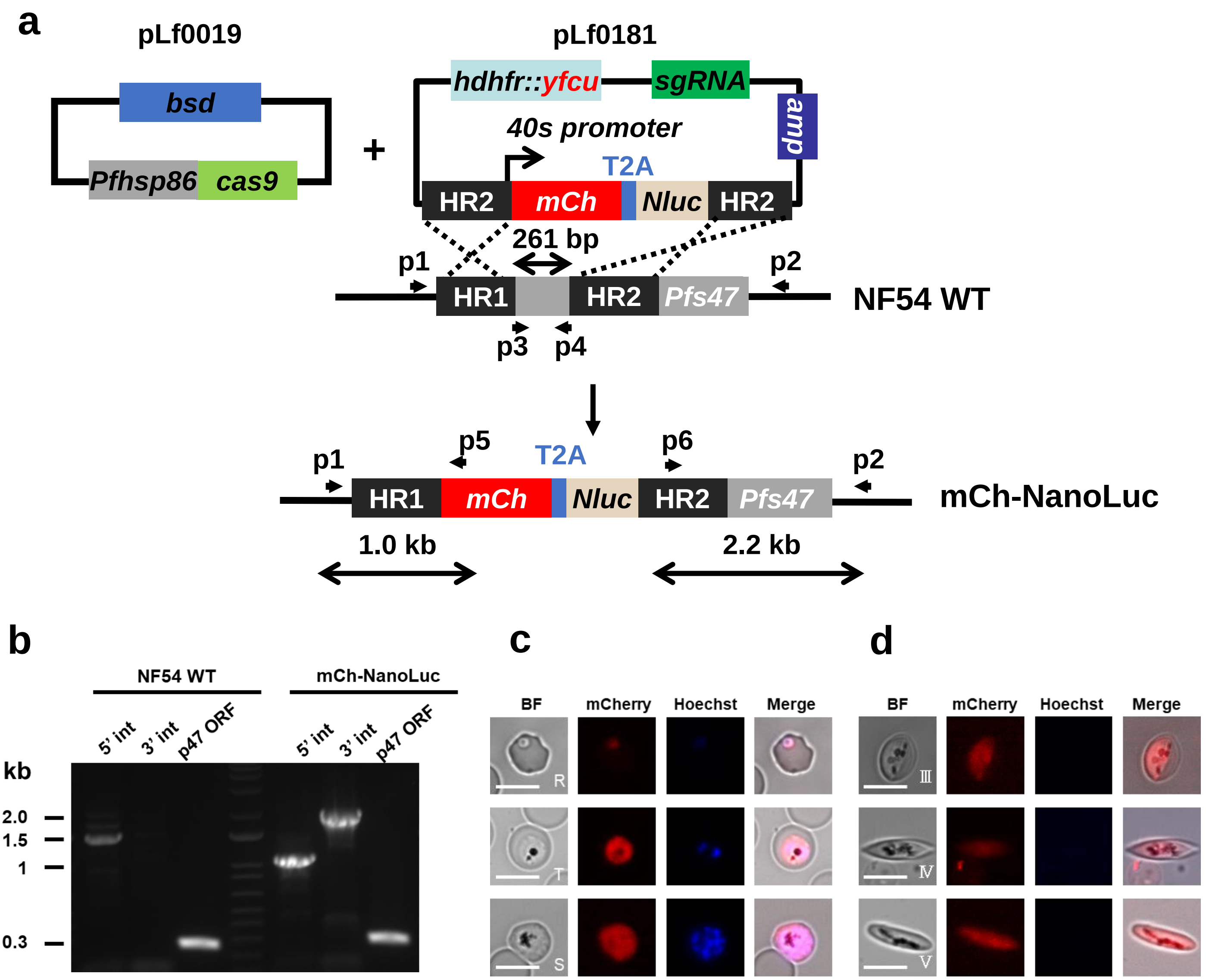

Supplementary Figure 1. Generation of *P. falciparum* NF54 reporter lines expressing mCherry and NanoLuc under the control of a 40S promoter. (a) Schematics of the CRISPR plasmids used for the generation of the mCherry-NanoLuc reporter line. The Cas9 expression plasmid (pLf0019) and donor DNA/gRNA plasmids (pL0181) constructs were used to introduce the mCherry-NanoLuc expression cassette with T2A into the Pf NF54 *p47* gene locus. The *p47* homology regions (HR1, HR2) used to introduce the donor DNA, location of primers (p) and PCR amplicons (in black) are indicated. Primer sequences (shown in black and bold) are shown in **Supplementary Table 1**. WT, wild-type; bsd, blasticidin selectable marker (SM); hdhfr::yfcu, SM in donor plasmid. **(b)** Genotyping analysis confirming the correct integration of the donor plasmids into the genome of the uncloned mCherry-NanoLuc reporter line (Exp221, 5-Int; primers p1/p5 for 1,087 bp, 3-Int; primers p6/p2; 2,188 bp). Primer positions and the expected DNA sizes are shown in (A), and primer sequences are shown in Table S1. The PCR product of the WT *p47* gene amplified by p3/p4 primers was detected in both WT and mCherry-NanoLuc reporter line, showing the mixed population with WT and the integrant. The weak 1.5 kb band with the 5'-int-primers is a non-specific fragment which is only present in the WT and not in the transgenic line. **(c, d)** Representative fluorescence microscopy pictures of live mCherry-NanoLuc line in the asexual blood stages **(c)** or gametocyte stages III, IV and V **(d)**. R, rings; T, trophozoites; S, schizonts; Nuclei were stained with Hoechst-33342. All pictures were recorded with standardised exposure/gain times to visualise differences in fluorescence intensity [GFP 0.7 s or mCherry 1s; Hoechst 0.2 s; bright field 0.1 s (1× gain)]. Bright field (BF) Scale bar, 7 µm.

a

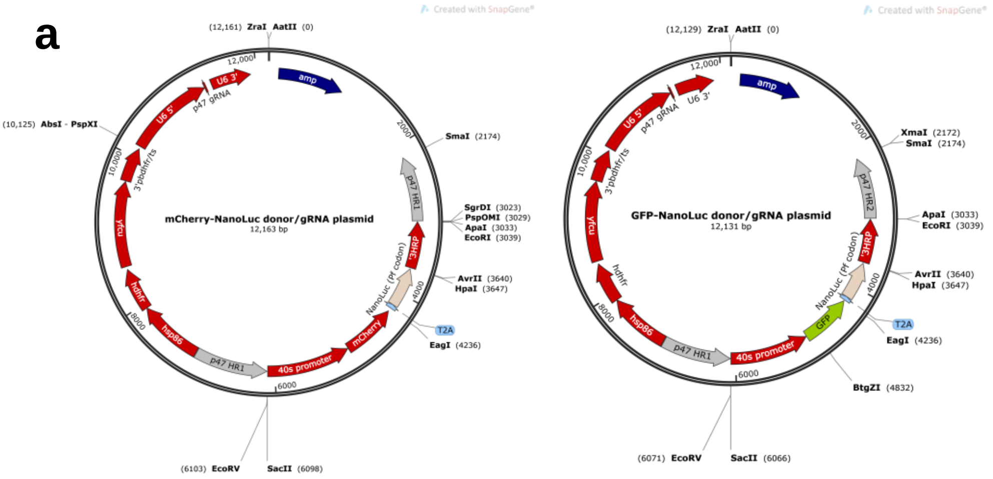

pLf0181
mCherry-NanoLuc donor/gRNA

pLf0182
GFP-NanoLuc donor/gRNA

b

TCCGGCGAGGGCGAGGGCGATGCCACCTACGGCAAGCTGACCCTGAAGTTCATCTGCACCACCGGCAAGC
TGCCCGTGCCCTGGCCCACCCTCGTGACCACCCTGACCTACGGCGTGCAGTGCTTCAGCCGCTACCCCGA
CCACATGAAGCAGCACGACTTCTTCAAGTCCGCCATGCCCGAAGGCTACGTCCAGGAGCGCACCATCTTCT
TCAAGGACGACGGCAACTACAAGACCCGCGCCGAGGTGAAGTTCGAGGGCGACACCCTGGTGAACCGCAT
CGAGCTGAAGGGCATCGACTTCAAGGAGGACGGCAACATCCTGGGGCACAAGCTGGAGTACAACTACAAC
AGCCACAACGTCTATATCATGGCCGACAAGCAGAAGAACGGCATCAAGGTGAACTTCAAGATCCGCCACAA
CATCGAGGACGGCAGCGTGCAGCTCGCCGACCACTACCAGCAGAACACCCCCATCGGCGACGGCCCCGT
GCTGCTGCCCGACAACCACTACCTGAGCACCCAGTCCGCCCTGAGCAAAGACCCCAACGAGAAGCGCGAT
CACATGGTCCTGCTGGAGTTCGTGACCGCCGCCGGGATCACTCTCGGCATGGACGAGCTGTACAAGCGGC
CGGTCGACGGAGAAGGAAGAGGAAGTTTATTAACATGTGGAGATGTAGAAGAAAATCCAGGACCAATGGTT
TTCACTTTAGAGGATTTCGTTGGAGACTGGCGTCAGACTGCTGGATATAATTTGGATCAGGTATTGGAACAG
GGAGGAGTGTCTAGTTTGTTTCAGAATCTTGGTGTGTCAGTTACTCCTATACAAAGAATTGTACTTTCTGGTG
AGAATGGATTAAAGATTGATATACATGTAATTATTCCTTACGAGGGATTAAGTGGAGATCAGATGGGTCAGAT
TGAAAAGATATTTAAGGTGGTATATCCAGTGGACGATCACCACTTTAAGGTTATTTTGCACTACGGAACTTTA
GTGATTGACGGTGTTACCCCAAACATGATTGATTATTTTGGTAGGCCATACGAGGGTATTGCAGTATTTGAT
GGTAAGAAAATTACAGTGACAGGAACACTTTGGAACGGAAACAAGATAATAGACGAGAGGTTGATAAATCCT
GATGGAAGTTTATTGTTCAGAGTGACTATAAACGGTGTGACCGGTTGGAGGTTATGCGAAAGGATATTAGCC
TAATCTAGAGTTAACCTAGGAGGAGG

Supplementary Figure 2. Maps of plasmids and sequence of the synthesised DNA used in this study. (a) Maps of plasmids (pLf0181 and pLf0182) used to generate the two transgenic reporter lines are shown. Refer to the Materials and Methods section for a description and details on plasmid generation**. (b)** The complete sequence of the synthesised GFP-T2A-NanoLuc DNA. Green; GFP. Yellow; T2A skip peptide. Gray; *P. falciparum* codon-optimised NanoLuc.

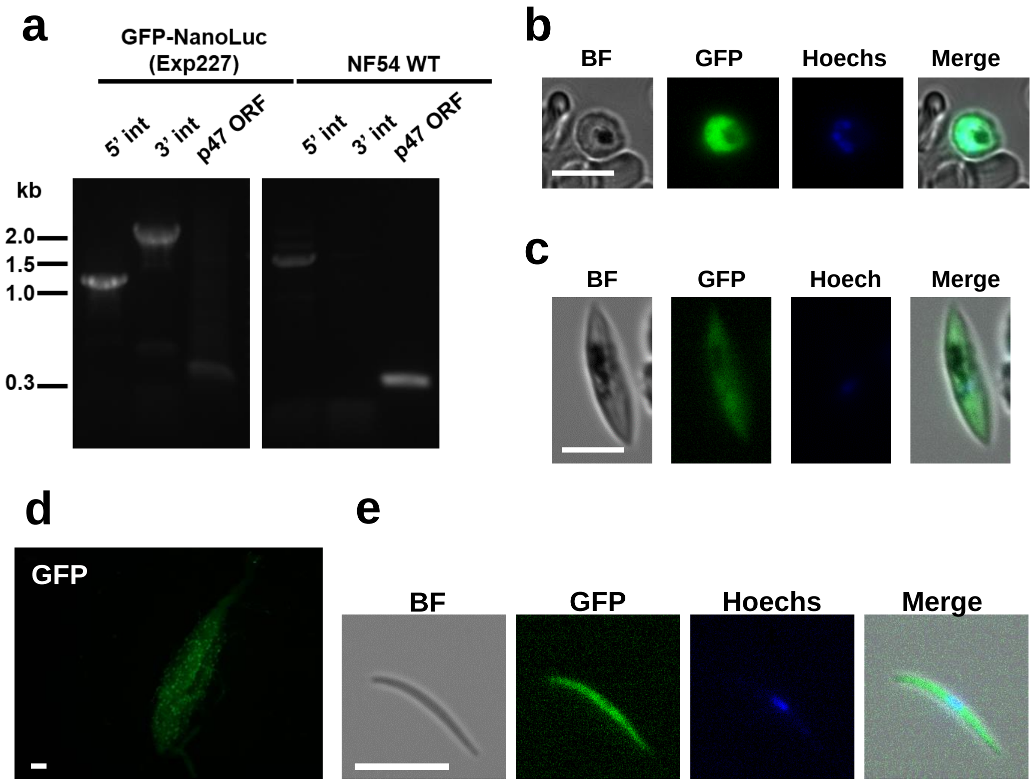

Supplementary Figure 3 Characterisation of the GFP-NanoLuc reporter line (Exp227) generated by independent transfection. (a) Genotyping analysis confirming the correct integration of the donor plasmids into the genome of the uncloned GFP-NanoLuc reporter line (Exp227, 5-Int; primers p1/p5 for 1,087 bp, 3-Int; primers p6/p2; 2,188 bp). Primer positions and the expected DNA sizes are shown in **Fig.1a**, and primer sequences are shown in Table S1. The PCR product of the WT *p47* gene amplified by p3/p4 primers was detected in both WT and GFP-NanoLuc reporter line, showing the mixed population with WT and the integrant. The weak 1.5 kb band with the 5'-int-primers is a non-specific fragment which is only present in the WT and not in the transgenic line. **(b)** Representative fluorescence microscopy images of live GFP-NanoLuc parasites in asexual blood stages. Nuclei were stained with Hoechst-33342. The images were obtained under standardised exposure/gain times to visualise differences in fluorescence intensity [GFP 0.7 s; Hoechst 0.2 s; bright field 0.1 s (1× gain)]. Live imaging analysis was performed at least thrice. Bright field (BF). Scale bar, 7 μm. **(c)** Representative fluorescence microscopy images of live GFP-NanoLuc line parasites in gametocyte stage. Nuclei were stained with Hoechst-33342. The images were captured with standardised exposure/gain times to visualise differences in fluorescence intensity [GFP 0.7 s; Hoechst 0.2 s; bright field 0.1 s (1× gain)]. Live imaging analysis was performed at least thrice. Bright field (BF) Scale bar, 7 μm. **(d)** Representative fluorescence microscopy pictures of *Anopheles* mosquito midgut infected with the GFP-NanoLuc reporter line. The oocysts in the complete midgut are shown as puncta in the image. Live imaging analysis was performed at least thrice. Scale bar, 40 μm. **(e)** Representative fluorescence microscopy images of live salivary gland sporozoites of the GFP-NanoLuc reporter line isolated on day 24 after mosquito infection. Nuclei were stained with Hoechst-33342. Bright field (BF). Scale bar, 7 μm.

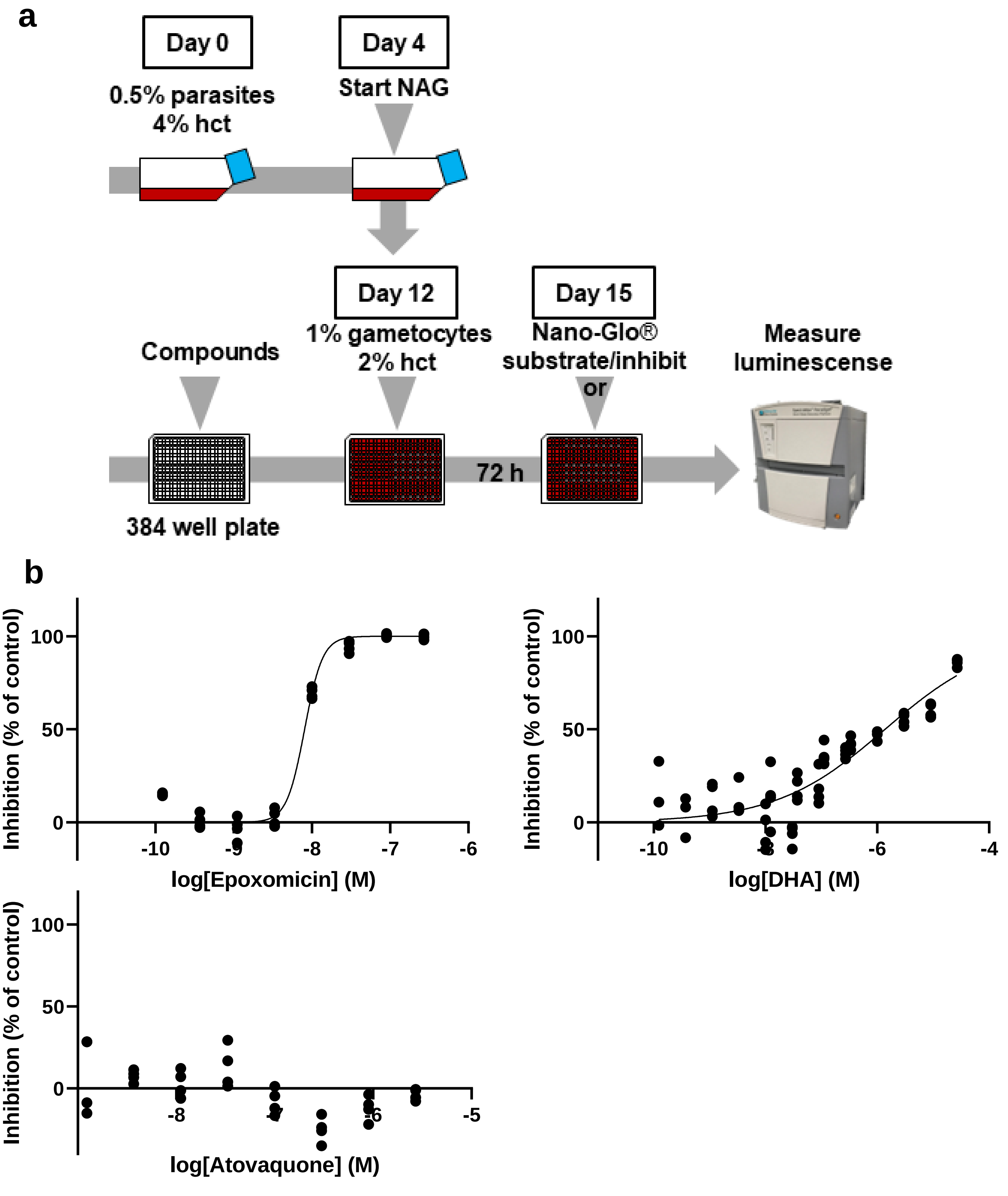

Supplementary Figure 4. Establishment of drug assay against gametocyte-stage parasites in a 384-well format. (a) Schematics of gametocyte stage drug assay in a 384-well format using the GFP-NanoLuc reporter line. Chemical compounds were dispensed in 384-well white plates, and day 12 gametocyte cultures were added to each well. After 72 h of incubation at 37 °C, the extracellular NanoLuc inhibitor and the substrates were added for NanoLuc reaction. **(b)** Dose-response curves of epoxomicin and DHA, validated anti-malarial compounds. The GFP-NanoLuc reporter lines were cultured in different concentrations of indicated anti-malarial compounds on 384-well plates. 270 nM epoxomicin and DMSO were used as positive and negative controls, respectively. The IC_{50} of epoxomicin and DHA was determined via non-linear regression from technical quadruplicate. The IC_{50} value of each anti-malarial compound is summarised in **Table 2**.

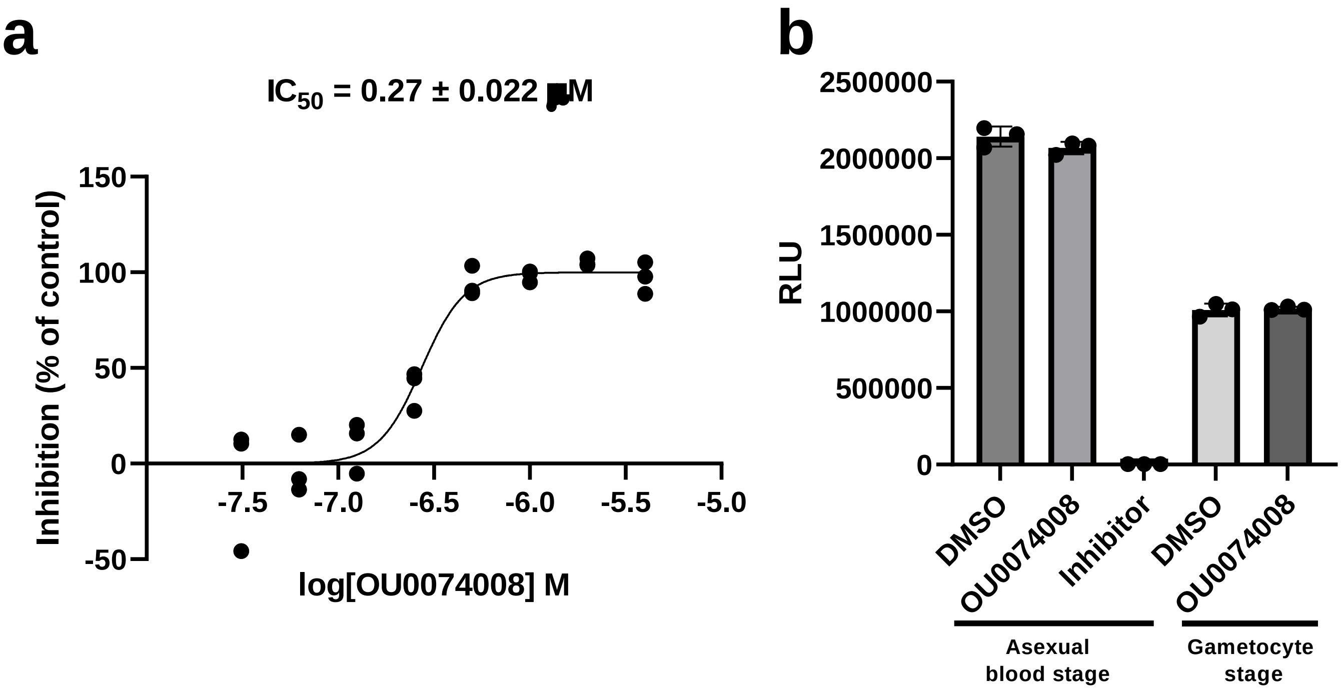

Supplementary Figure 5. LDH assay and NanoLuc inhibiton assays using OU0074008. (a) Evaluation of anti-malarial activity of OU0074008 by LDH assay. The GFP-NanoLuc reporter lines were cultured in different concentrations of OU0074008. After 72 h incubation, LDH assay was performed to determine the IC_{50} value of OU0074008 against the asexual blood stage. 1 µM DHA and DMSO were used as positive and negative controls, respectively. The IC_{50} of OU0074008 was determined via non-linear regression from technical triplicates. **(b)** Inhibition of NanoLuc was measured by adding the OU0074008 compound to the whole lysates from GFP-NanoLuc line in either asexual blood or gametocyte stage. NanoLuc inhibitor provided by Promega was used as a positive control for inhibition, which shows almost complete inhibition of NanoLuc (RLU ; 3663±273). Means and SD from technical triplicate are shown as box bars and error bars, respectively.

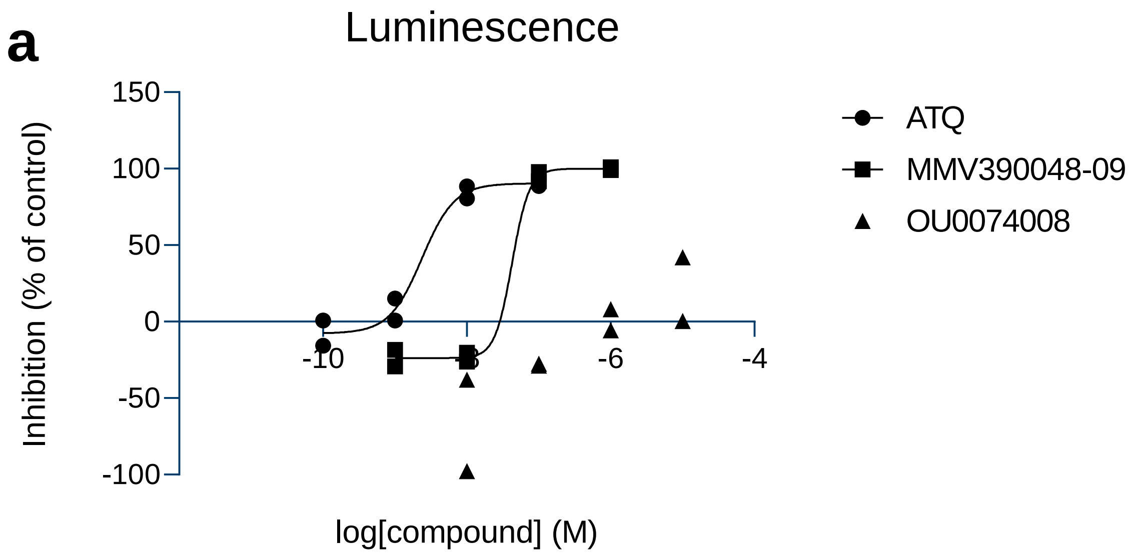

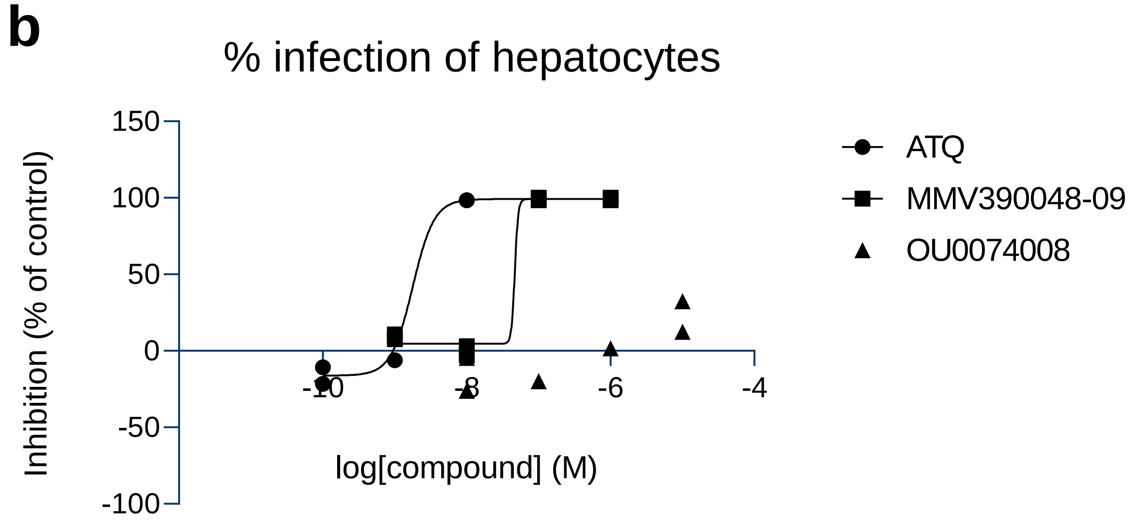

Supplementary Figure 6. Evaluation of antimalarial drug efficacy of OU0074008 at the liver stage. (a, b) The effect of atovaquone, MMV390048 and OU0074008 on intrahepatic parasite development was assessed either by luminescence (**a**) or imaging of HSP70 positive forms (**b**) on day 4 post infection. 100 nM MMV390048 and DMSO were used as positive and negative control, respectively. Symbols indicate the values from technical duplicates per dilution.

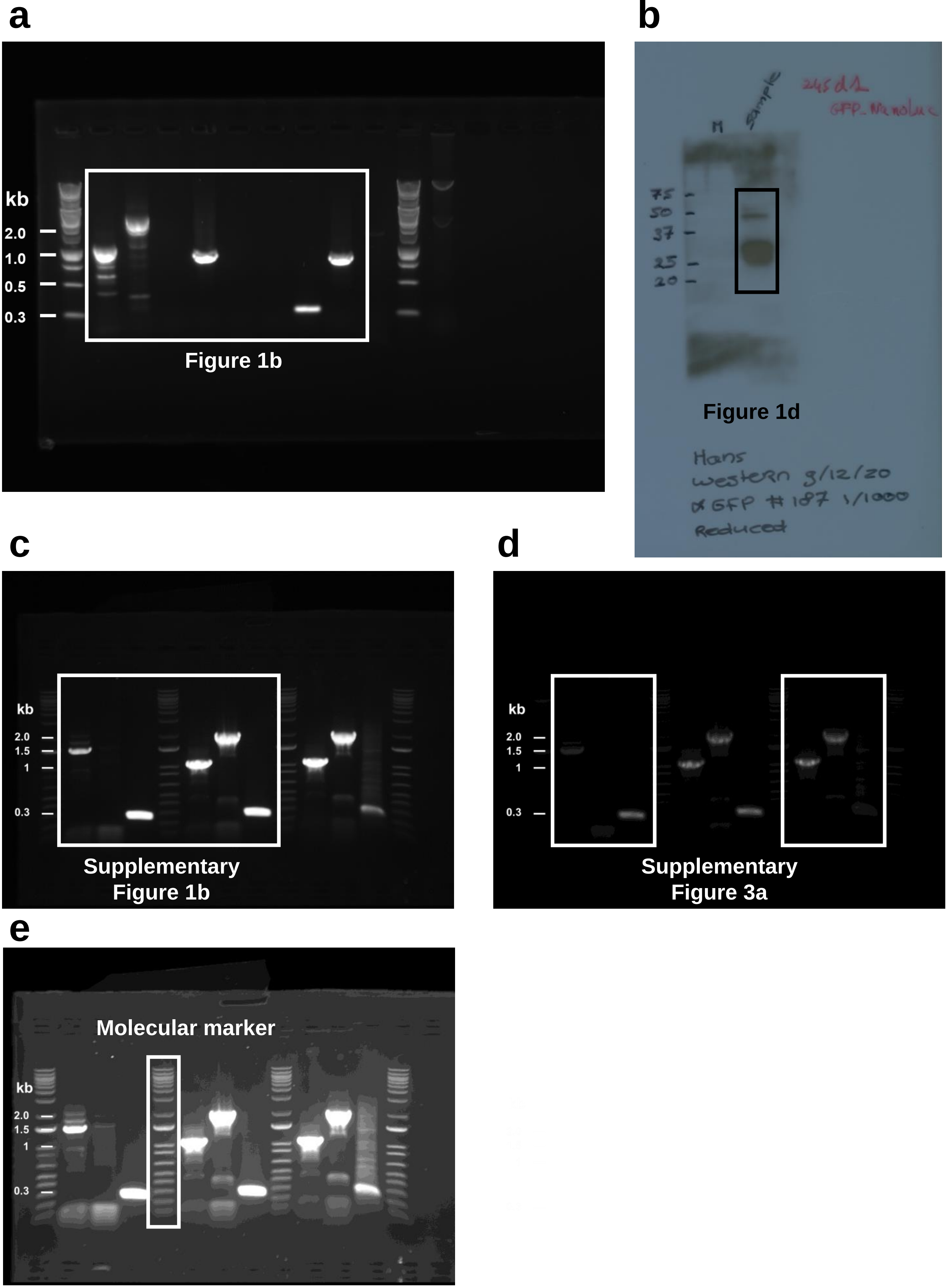

Supplementary Figure 7. Original images of agarose gel electrophoresis and Western blotting used in Figure 1 and Supplementary Figures 1 and 3 . (a) The images of agarose gel used in Figure 1b (**b**) The images of agarose gel used in Figure 1d. (**c, d**). The image of agarose gel used in Supplementary Figures. **(e)** The agarose gel pictures in (**c,d**) are exposed for a long time to clearly indicate the molecular marker.

Primer name	Primer ID	Sequence	Enzyme	Description
**Genotyping**				
5-Int F	P1	CATTCCTAACACATTATGTGTATAAC		Genotyping to confirm donor plasmid integration
3-Int R	P2	CATCGAAATGCGTATTAATATGAC		Genotyping to confirm donor plasmid integration
p47 ORF F	P3	AACTATTAAGCTCAACACAATACG		Genotyping to confirm the absence of WT population
p47 ORF R	P4	CTAAATGATATGCGCTGGAATC		Genotyping to confirm the absence of WT population
5-Int R	P5	AATGATCACATGATGGATTGATATTG		Genotyping to confirm donor plasmid integration
3-Int F	P6	ATAGCTCGAGGTTAACCTAGGGAAGTATATGAG		Genotyping to confirm donor plasmid integration
GLURP.F	P7	TGAATTTGAAGATGTTCACACTGAAC		Positive control for PCR amplification of glurp gene
GLURP.R	P8	GTGGAATTGCTTTTTCTTCAACACTAA		Positive control for PCR amplification of glurp gene
**Plasmid construction**				
GFP-T2A-NanoLuc.F	P9	TCCGGCGAGGGCGAGGGCGATGCCA	BtgZI	Amplification of the GFP-T2A-NanoLuc sequence
NanoLuc.R	P10	CATATACTTCCCTAGGTTAACTCTAGATTAGGCTAATATCC	AvrII	Amplification of the GFP-T2A-NanoLuc/NanoLuc sequence
NanoLuc.F	P11	TGCAAGCTTGCGGCCGGTCGACGGAGAAGGAAGA	EagI	Amplification of NanoLuc sequence to replace Firefly luciferase

Supplementary Table 1. Primers used in this study
