# Supplementary material for: A versatile Plasmodium falciparum reporter line expressing NanoLuc enables highly sensitive multi-stage drug assays
Source: Commun Biol. 2023 Jul 12;6:713. doi: 10.1038/s42003-023-05078-5 (PMC10338434; doi:10.1038/s42003-023-05078-5)
Supplement: Supplementary file 3 — Description of Additional Supplementary Files [file 42003_2023_5078_MOESM3_ESM.pdf]

Description of Additional Supplementary Files

File name: Supplementary Data
Description: Source data behind the graphs in the paper.
